# Response of rhizosphere microbial community of Chinese chives under different fertilization treatments

**DOI:** 10.3389/fmicb.2022.1031624

**Published:** 2022-11-21

**Authors:** Tianhang Niu, Jianming Xie, Jing Li, Jing Zhang, Xiaodan Zhang, Hongyan Ma, Cheng Wang

**Affiliations:** ^1^College of Horticulture, Gansu Agricultural University, Lanzhou, China; ^2^Lanzhou New Area Agricultural Science and Technology Development Co., Ltd., Lanzhou, China

**Keywords:** Chinese chives, slow-release fertilizer, microbial diversity, rhizosphere, conventional fertilizer

## Abstract

Soil microorganisms play an irreplaceable role in agricultural production, however, an understanding of response of soil microorganisms to slow-release and common fertilizer applications is limited. In this study, different amounts of slow- release fertilizer were used to overwintering Chinese chives growing area in a plastic greenhouse to investigate the effects of on rhizosphere soil physicochemical properties and soil microbial communities (bacteria and fungi) of Chinese chives. The result displayed that application of slow-release fertilizer significantly improved soil nutrients, soil enzyme activity, and soil microbial community structure and diversity compared to conventional fertilizer application. Compared with T1 treatment, the content of total nitrogen (TN) and available phosphorus (AP), and the SU-E activity in the soil of T2 (NPK: 62.8 kg · 667 m^-2^) increased by 42.58%, 16.67%, and 9.70%, respectively, showing the best effects. In addition, soil bacterial diversity index and soil microbial community structure were improved as indicated by increased relative abundance of each species, such as *Byssovorax*, *Sandaracinus*, and *Cellvibrio*. Oppositely, the both soil fungal diversity and the number of species decreased after fertilizationthe relative abundance of Ascomycota increased in each fertilization treatment detected by ITS sequencing. Further, the relative abundance of pathogenic fungi such as *Pezizomycetes*, *Cantharellales*, and *Pleosporales* decreased in the T2 treatment. Principal Coordinates Analysis (PCoA) showed that both the amount of fertilizer applied and the type of fertilizer applied affected the soil microbial community structure. RDA evidenced that soil bacteria, Proteobacteria and Gemmatimonadetes, were closely correlated with soil AN, SOM, and AK. Acidobacteria were closely correlated with soil pH, TN, and AP. Ascomycota was closely correlated with soil pH and TN. In conclusion, the application of slow-release fertilizers and reduced fertilizer applicationcould improve soil physical and chemical properties as well as soil microbial community structure and diversity, contributing to sustainable soil development. The recommended fertilization rate for overwintering Chinese chives is NPK: 62.8 kg · 667 m^−2^.

## Introduction

The rhizosphere is the main microbial activity region in the soil environment (Kuzyakov and Razavi, 2019). It refers to the narrow soil area attached to and influenced by the roots of plants and their secretions; various complex biological and biochemical processes occur in this area (Soni et al., 2017; Kuzyakov and Razavi, 2019). Previous studies have shown that fertilizer application can improve soil physical structure, nutrients and alter the diversity of soil microorganisms (Ye et al., 2019; Lu et al., 2020). Microbial community functional diversity reflects the soil microbial ecological function (Xiao et al., 2016). Changes in microbial community composition affect soil functional processes and attributes (Zhao M. et al., 2014; Bell and Tylianakis, 2016). Therefore, soil microorganisms play a key role in the plant–soil system (Li et al., 2022).

Fertilization is an indispensable agronomic measure in crop production, promoting crop growth to a certain extent, ensuring crop yield, and improve economic benefits (Gao et al., 2015; Rashidzadeh et al., 2015). However, in actual agricultural production, excessive chemical fertilizers application has been increasing. When fertilizer input is exceptionally large, it can easily cause soil nutrient accumulation, change soil physical and chemical properties, and decrease soil organic matter content (Beauregard et al., 2010; Qiao et al., 2016). Meanwhile, studies have shown that excessive use of nitrogen reduces the diversity of soil microbial communities and simplifies their structure (Zhao M. et al., 2014; Wu L. et al., 2021; Qu et al., 2022), conversely affecting the metabolic activity of microorganisms (Wu et al., 2020). When the soil microbial community structure is unbalanced, the possibility of plants contracting soil-borne diseases rise (Fuchslueger et al., 2014). Long-term localization experiments found that soil physical and chemical properties varied after long-term fertilization, resulting in changes in soil microbial community structure (Zhao J. et al., 2014; Zhou et al., 2015). For example, long-term fertilization decrease the relative abundance of soil Proteobacteria, Bacteroidetes, and Actinobacteria (Rousk et al., 2010). In contrast, the relative abundance of Acidimicrobiia, Alphaproteobacteria, Chloroflexi, and Gammaproteobacteria in the soil increased with the growth of nitrogen content in the soil (Zhou et al., 2015). Studies on fungi have shown that long-term nitrogen application decreasedsoil fungal diversity (Weber et al., 2013; Sun et al., 2014), and increasing the relative abundance of the harmful fungi *Chaetothyriales*, *Pleosporaceae*, *Bipolaris*, and *Cyphellophora* (Ma et al., 2018).

To overcome the above problems arising from the application of common chemical fertilizers in agricultural production, agriculturalists have focused their attention on slow/controlled-release fertilizers. As a new type of fertilizer, the characteristic of slow/controlled-release fertilizer is that it can maintain fertilizer efficiency for a long time, reduce the frequency of fertilization (Wang et al., 2020), as well as increase crop yields, economic benefits, and improve fertilizer utilization (Gao et al., 2015; Teng et al., 2018). Zhu et al. (2012) reported that applying controlled-release urea in tomato cultivation could promote plant growth and increase leaf chlorophyll content. Further, applying slow/controlled-release fertilizer can increase the content of available soil nutrients and improve the physical and chemical properties of soil (Yang et al., 2016). Meanwhile, using slow/controlled-release fertilizers can improve soil microbial diversity (Hayatsu, 2014). After applying urea-formaldehyde fertilizer to the onion and sugar beet plantings, soil microbial diversity was altered, with the relative abundance of a few species increased or decreased (Ikeda et al., 2014). The distribution, structure, and diversity of rhizosphere soil microbial communities of rice improved after the deep application of slow-release fertilizer to the rice cultivation process (Chen et al., 2022). After the application of slow-release fertilizer in the cultivation of green pepper, the number of bacteria and fungi was significantly higher in all fertilization treatments than in the blank treatment, the results showed that the application of slow-release fertilizer increased the number of soil microorganisms (Teng et al., 2018). Studies have also shown that the application of encapsulated slow-release fertilizers can also increase the functional diversity of soil bacteria (Fertahi et al., 2021). Similarly, studies on oilseed rape (*Brassica napus L.*) have shown that bacterial communities have a higher capacity for nutrient metabolic cycling and improved bacterial nitrification after the application of slow-release fertilizers (Liao et al., 2020).

Chinese chives have a long cultivation history in China. During the growth of chives, rhizosphere soil microorganisms break down various nutrients in the soil for chives to grow and help the plant resist diseases. Previous trials have shown that Chinese chives yields significantly improved with the application of slow-release fertilizers (Niu et al., 2020). However, studies revealing the effects of slow-release fertilizer on the soil environment during chives cultivation are limited. Therefore, the is study aimed to investigate the effect of slow-release fertilizer application on the composition of rhizosphere microbial communities using high-throughput sequencing and to improve the understanding of the effect of slow-release fertilizer application on soil microbes during Chinese chives cultivation. The specific aims were investigate (1) the effects of different fertilizer application treatments on soil physicochemical properties and soil enzyme activities, (2) the response of soil microbial (bacterial and fungal) diversity and community structure to different fertilization treatments, and (3) the relationship between soil microbial communities and soil physicochemical properties and soil enzyme activities under different fertilizer application treatments.

## Materials and methods

### Field experiment

The experiment was conducted from June 2017 to February 2019 in the Qingchi village, Wushan County, China (N 34^°^25″ –34°57″, E 104°34″ –105°08″), which is a traditional Chinese chive growing area. The soil type in the area was fluvisols (IUSS Working Group WRB, 2015). The study area is located in a river valley with a flat topography, an average annual temperature of 9.6°C and uniform soil fertility. The size of the test site was 56 m × 18 m. Conventional chemical methods determined the soil nutrient content of the tested site (Gong et al., 2019): alkali-hydrolyzable N, 78.98 mg · kg^−1^; available P, 190.98 mg · kg^−1^; available K, 244.87 mg · kg^−1^; soil organic matter, 20.48 g · kg^−1^; and pH, 7.7.

Chinese chive (cv. “Chive God F1”) seeds were obtained from the Fugou County Seedling Research Institute in Henan, China. The chives were planted on June 20, 2017. The upper part of the leek leaves and fibrous roots were cut before planting to promote new root development. They were planted at a row spacing of 20 cm and a hole spacing of 10 cm, with three chives per hole. The slow-release fertilizer (SRF), a resin-coated compound fertilizer, used in the experiment was provided by Hubei Ezhong Ecological Engineering Co., Ltd., Hubei Province, China. The SRF had a release longevity of 120 days and an NPK ratio of 26: 11: 11. Conventional fertilizers used in this experiment included urea containing 46% N, calcium superphosphate containing 12% P, and potassium sulfate containing 51%.

Five treatments were established as randomized complete block designs, with three replicates at the Qingchi Chinese chives in Wushan County, China. The fertilization trial was conducted for two consecutive years at this test site. Specific fertilizer application rates were shown in Table 1. The T1 treatment was fertilized according to the traditional and customary fertilizer dosage used by local farmers. The T3 treatment replaced the common fertilizer used in the T1 treatment with an equal amount of slow-release fertilizer. The T2 treatment was fertilized according to the nutrient balance method that requires absorption of N 1.7 kg, P 0.5 kg, and K 1.8 kg per 1,000 kg of Chinese chives produced. As a result, N, P, and K utilization rates were 35, 25, and 50%, respectively. The T4 treatment replaced the slow-release fertilizer in the T2 treatment with an equal amount of common fertilizer. The details are as follows: three test plots (as three replicates) were set up for each treatment, and each trial plot had an area of 50 m^2^. Each experimental plot was randomly distributed. The management measures were identical among the experimental plots except for the different fertilizer applications. In this study, the target yield of Chinese chives was 3,000 kg · 667 m^−2^, and 14.6 kg · 667 m^−2^ of N, 6 kg · 667 m^−2^ of P, and 10.8 kg · 667 m^−2^ of K were applied, respectively. Therefore, the total fertilizer application for the T2 treatment decreased by 31.22% compared to the total fertilizer application for the T1 treatment, in which N decreased by 33%, P decreased by 68%, and K increased by 110%. Insufficient application of P and K in the T2 and T3 treatments was supplemented using calcium superphosphate and potassium sulfate. The above calculation of fertilizer application rate has been reported in earlier studies (Wang et al., 2020).

**Table 1:** The amount of fertilizer applied to each treatment.

Treatments	Amount of fertilization				
	N/(kg·667 m^−2^)	P_2_O_5_/(kg·667 m^−2^)	K_2_O/(kg·667 m^−2^)	Total fertilization /(kg·667 m^−2^)	Compared with T1 ± %
CK	0	0	0	0	–
T1	43.7	37.3	10.3	91.3	–
T2	29.2	12.0	21.6	62.8	−31.22%
T3	43.7	37.3	10.3	91.3	0
T4	29.2	12.0	21.6	62.8	−31.22%

CK, no fertilizer application; T1, conventional fertilizer application; T2, reduced fertilizer application for slow-release fertilizer; T3, conventional fertilizer application for slow-release fertilizer; T4, reduced fertilizer application for conventional fertilizer.

Treatments T1 and T4 were applied on July 5, July 22, August 9, and August 28, 2017, with four fertilizer treatments totaling 50% of the total fertilizer applied that year. On April 18, July 26, and September 25, 2018, the total amount of fertilizer applied in three applications was 50% of the total fertilizer applied in the year. The remaining 50% was fully applied on November 20, 2017. Treatments T2 and T3 were applied on July 5, 2017 (50% of the total fertilizer applied that year), April 18, 2018 (25% of the total fertilizer applied in the year), and July 26, 2018 (25% of the total fertilizer applied in the year) as a follow-up. The remaining 50% was fully applied on November 13, 2018. Management practices were strictly consistent among treatments except for fertilizer application.

### Soil sampling

Soil samples were collected in January 2019 (60 days after fertilization). Soil samples were randomly collected from nine points in each treatment, uniformly mixed and divided into three replicate samples; 15 samples were obtained from five treatments in this experiment. This was done as follows: nine plots of equal size were randomly selected from each treatment plot (avoiding the treatment edges), the top 0–5 cm of soil was removed, and the plants and soil (overall thickness of about 20 cm) were divided as a whole from the planting site, placed on sterile paper, larger soil blocks around the plant roots were removed, rhizosphere soil was gently brushed off with a brush, collected in sterile lyophilization tubes, and immediately stored in an ice box (Semenov et al., 2019). When samples were collected between different treatments, the residues attached to the shovel were cleaned with sterile paper and the shovel was disinfected to avoid contamination between treatments. The rhizosphere soil was divided into two parts, one for soil microbiological analysis and the other was passed through a 2 mm sieve to remove residual roots and gravel, air dried and used to determine the physical and chemical properties of the soil.

### Determination of soil physical and chemical properties

To determine soil physical and chemical properties, we ground the air-dried soil samples and passed them through a 2.0 mm sieve before assessing their soil nutrient content. Soil pH was measured at a water: soil ratio of 1: 2.5. The total nitrogen (TN) was determined using the Kjeldahl method by an automatic Kjeldahl analyzer K1100F device (Jinan Hannong Instrument Company, Jinan, China; Wang et al., 2020). Soil organic matter (SOM), alkali-hydrolyzable nitrogen (AN), available phosphorus (AP), and available potassium (AK) were determined according to the method described by Zhao M. et al. (2014) and Wang et al. (2021). Soil urease (S-UE), soil alkaline phosphatase (S-ALP), soil sucrase (S-SC), soil catalase (S-CAT), and soil polyphenol oxidase (S-PPO) activities were determined using Solarbio soil urease kit (Solarbio, BC0120), soil alkaline phosphatase kit (Solarbio, BC0280), soil sucrase kit (Solarbio, BC0240), soil catalase kit (Solarbio, BC0100) and soil polyphenol oxidase kit (Solarbio, BC0110). The above soil enzyme kits are manufactured by Solarbio (Beijing Solarbio Science & Technology Co., Ltd., Beijing, China). The specific methods and measurement procedures were carried out according to the instructions in the manual.

### DNA extraction, PCR amplifications, and Illumina library generation

Three rhizosphere soil samples per treatment, totaling 15 samples, were used in the study. Soil microbial DNA was extracted using the soil microbial genomic DNA extraction kit (Tiangen Biotech, Beijing, China), and the results of 1% agarose gel electrophoresis showed clear bands and good DNA quality.

PCR amplifications were performed using the KAPA HiFi HotStart ReadyMix PCR Kit (KAPA Biosystems, United States). The total volume of each reaction system was 25 μl, including 2 × KAPA HiFi HotStart ReadyMix, 25 μmol/l of each primer, 10 ng of DNA template, and PCR Grade Water. The universal primers for bacterial 16S rRNA amplification were B341F (5´-CCTACGGGNGGCWGCAG-3′) and B805R (5′- GGACTACVSGGGTATCTAAT-3′). The common primers for fungal ITS amplification were ITS3 (5′-GATGAAGAACGYAGYRAA-3′) and ITS4 (5′-TCCTCCGCTTATTGATATGC-3′). PCR amplification was performed under the following conditions: pre-denaturation at 95°C for 3 min. Denaturation was carried out at 95°C for 30 s, annealing at 55°C for 30 s, extension at 72°C for 30 s for eight cycles, and a final extension at 72°C for 5 min (Huang, 2018). DNA was stored at 4°C until analysis. Electrophoresis was performed with 2% agarose gels, and the cut gel was recovered according to the procedure of the QIAquick Gel Extraction Kit (Qiagen, Germany). Sequencing was performed using an Illumina MiSeq system (Illumina MiSeq, United States).

### Statistical analysis

After obtaining the raw data, data filtering was first required: using Mothur (1) (Schloss et al., 2009) to remove sequences with an average quality score of ≤ 20, to remove sequences containing N, and to remove sequences with excessively long homopolymers (>10 bp); (2) to remove sequences with excessive primer mismatches (≥4 bp) and to remove primer sequences (Schloss et al., 2009); (3) to remove sequences with excessively short (≤200 bp) and too long (≥500 bp) sequences (Schloss et al., 2009). In addition, (4) bacterial sequences were removed from chimera using UCHIME (Edgar et al., 2011) with the Gold dataset (Haas et al., 2011) as a reference, and fungal sequences were removed from chimera using UCHIME (Edgar et al., 2011) with the UNITE database (Koljalg et al., 2013) as a reference.

The bioinformatics analysis was based on the R.^1^ The dataset was analyzed using Mothur (Schloss et al., 2009). Use USEARCH software to cluster valid sequences into operational taxes based on the 97% similarity threshold. Mothur (v.1.30.1) calculated the relative abundance of rhizosphere microbial taxa, Chao1, ACE, Shannon, Simpson, and other alpha diversity indices. Bacterial sequences were systematically classified using the RDP classifier using the SILVA (SILVA 132) database as a reference for OTU sequences. Fungalsequences were systematically classified using the RDP classifier using the UNITE database (Koljalg et al., 2013) as a reference for OTU sequences. The OTU sequences were classified into phylotypes and matched to the SILVA database using PyNAST. The intercommunity distance matrix was generated using UniFrac, and the UniFrac matrix was subjected to principal coordinate analysis (PCoA). Data were analyzed using Statistical Analysis Software (SPSS software, 22.0, SPSS Institute Inc., United States), and treatment effects were determined using Duncan’s multiple range test (*p* < 0.05). Soil physicochemical properties, soil enzyme activity, and microbial characteristics of different fertilization treatments were analyzed by one-way analysis of variance (ANOVA) for data (*p* < 0.05). Correlations among the soil microbial compositions and soil properties activities were determined using redundancy analysis (RDA). The RDA was performed using the CANOCO 5 software package. Plotting was performed using Origin 2021. Sequences were uploaded to the National Center for Biotechnology Information (NCBI) Sequence Read Archive under BioProject PRJNA852613 and PRJNA852643.

## Results

### Change in physical and chemical properties under different fertilization treatments

**Figure 1:**
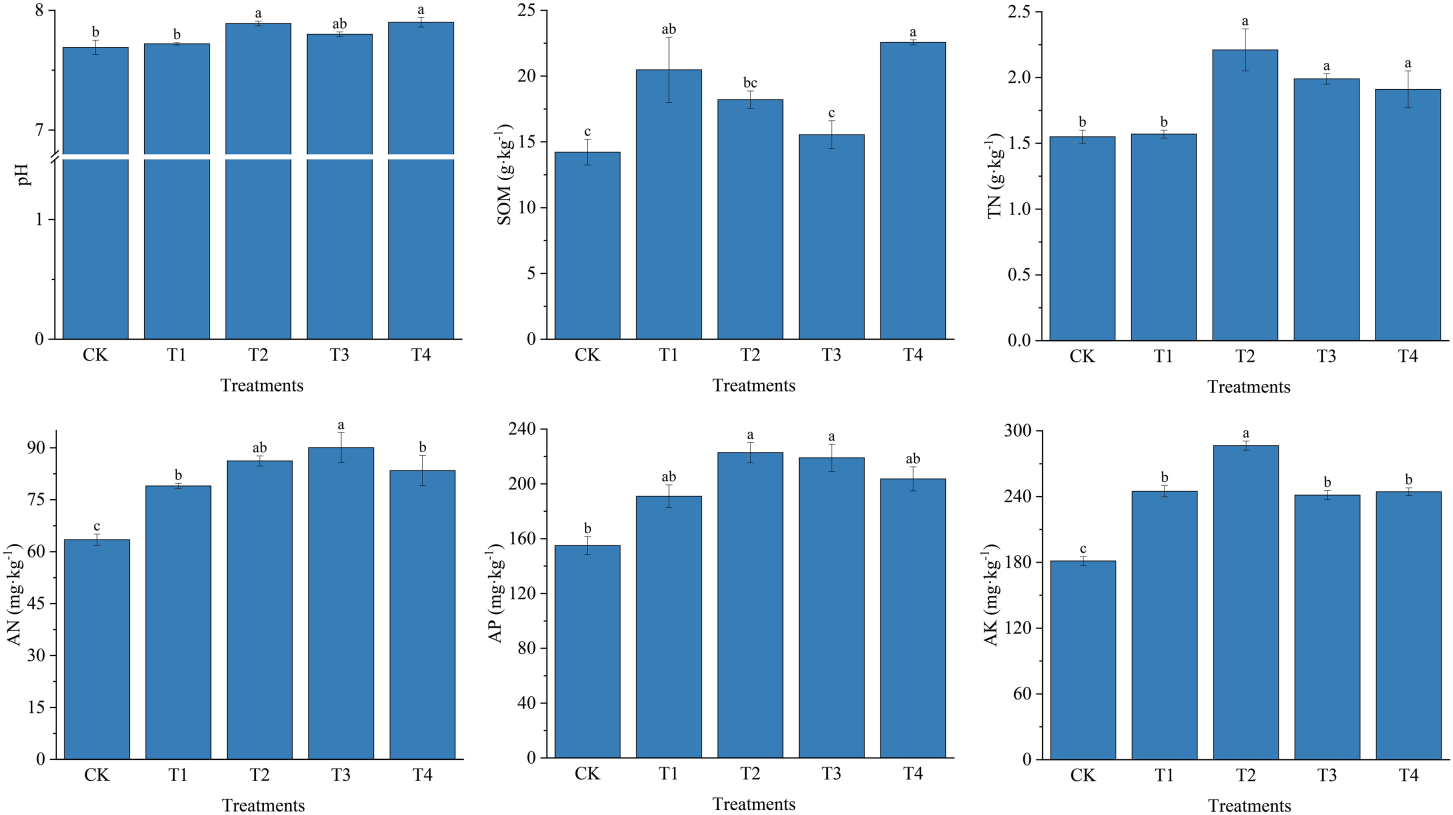
Physical and chemical properties of soil under different fertilization treatments. CK, no fertilizer application; T1, conventional fertilizer application; T2, reduced fertilizer application for slow-release fertilizer; T3, conventional fertilizer application for slow-release fertilizer; T4, reduced fertilizer application for conventional fertilizer. SOM, soil organic matter; TN, total nitrogen; AN, alkali-hydrolyzable Nitrogen; AP, available phosphorus; AK, available potassium. Values are reported as repeated mean ± standard error. According to Duncan’s test, the average of the different letters (such as a, b, and c) in each column was significantly different at *p* < 0.05.

Soil physicochemical properties as affected by fertilizer application are shown in Figure 1. Specifically, soil pH in the five treatments ranged from 7.69 to 7.90 after fertilization, with the highest pH in treatment T4. Fertilizer application increased soil TN, AN, AP, and AK contents overall (*p <* 0.05), ranging from 1.55 to 2.21 g · kg^−1^ for TN, 63.47 to 90.06 mg · kg^−1^ for AN, 155.01 to 222.81 mg · kg^−1^ for AP, and 181.28 to 286.52 mg · kg^−1^ for AK. TN increased by 15.71 and 26.76% in T2 and T3 treatments, respectively, compared to T4 and T1 treatments. Fertilizer application significantly increased TN and AN compared to CK. TN increased by 11.06 and 26.49% in T2 and T4 treatments compared to T3 and T1, respectively. The AK content of the T2 treatment was 17.00, 18.72, and 17.22% higher than theT1, T3 and T4 treatments, respectively. Different fertilizer treatments also affected soil organic matter content, which increased in each fertilizer treatment compared to CK. However, the soil organic matter content was higher in treatments with common fertilizers (T1 and T4) than in treatments with slow-release fertilizers (T2 and T3).

### Enzyme activity changes in soils with different fertilization treatments

**Figure 2:**
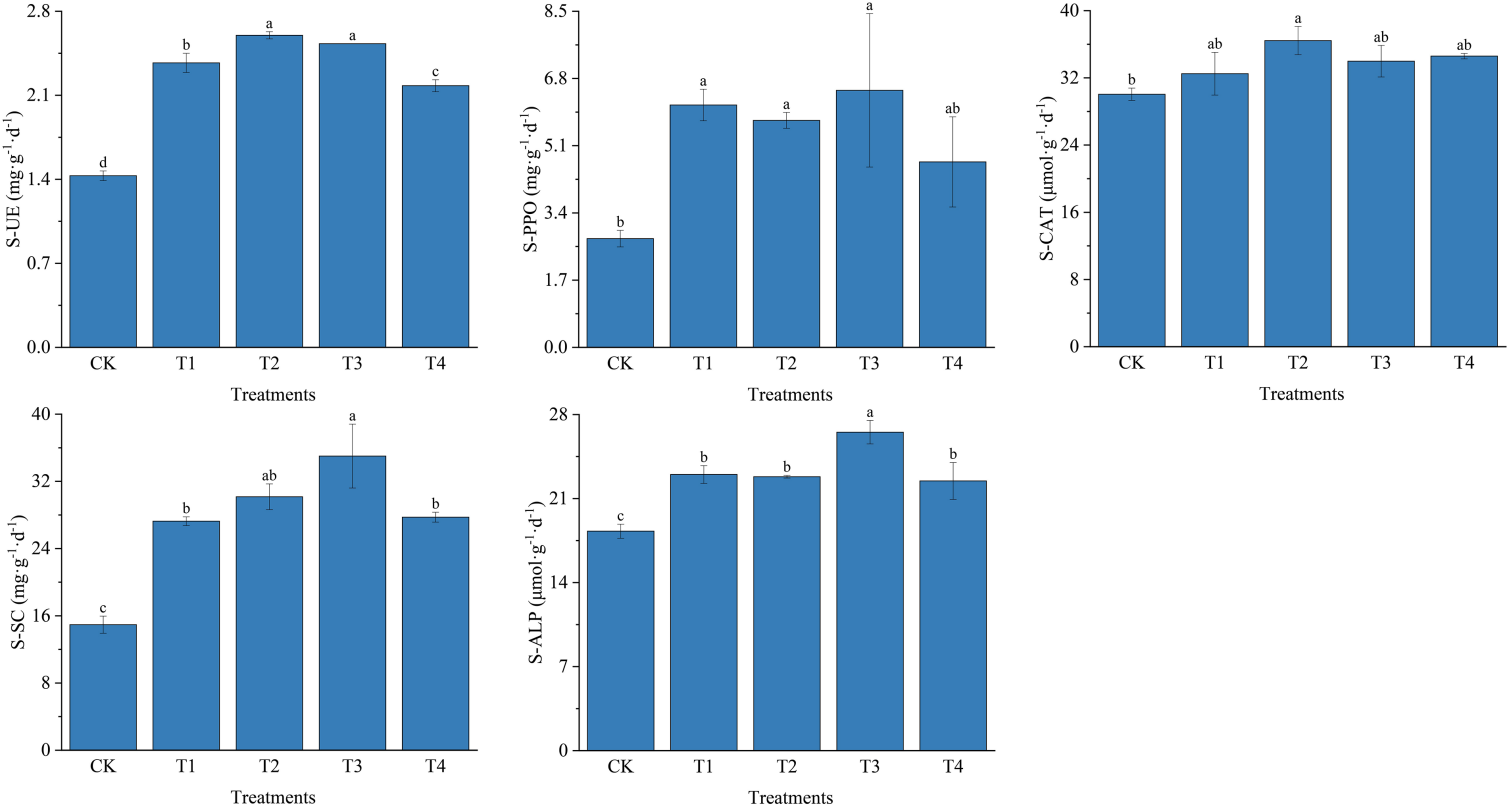
Soil enzyme activities under different fertilization treatments. CK, no fertilizer application; T1, conventional fertilizer application; T2, reduced fertilizer application for slow-release fertilizer; T3, conventional fertilizer application for slow-release fertilizer; T4, reduced fertilizer application for conventional fertilizer. S-UE, soil urease; S-PPO, soil polyphenol oxidase; S-CAT, soil catalase; S-SC, soil sucrase; S-ALP, soil alkaline phosphatase. Values are reported as repeated mean ± standard error. According to Duncan’s test, the average of the different letters (such as a, b, and c) in each column was significantly different at *p* < 0.05.

For soil enzyme activities, fertilization resulted in increased SU-E, S-PPO, S-CAT, S-SC, and S-ALP activities (Figure 2). The most significant changes in SU-E activity were observed under different fertilizer treatments. Compared to the T4 treatment, the T2 treatment S-UE activity was significantly higher by 19.27% (*p* < 0.05). SU-E activity was significantly increased by 6.75% (*p* < 0.05) in T3 treatment compared to T1 treatment. In contrast, different fertilization treatments influenced S-PPO activity at lower levels. However, in T1, T2, and T3 treatments, S-PPO activity was significantly higher than in CK. However, there were no significant differences between T1, T2, T3, and T4 treatments. Similar to the S-PPO activity, S-CAT activity was also influenced at a lower level by different fertilization treatments; the S-CAT activity was increased by 21.26% in T2 treatment compared to CK. Fertilizer application also had a positive effect on S-SC activity. S-SC activity was highest among fertilizer application treatments at T3 with 35.01 mg ·g^−1^·d^−1^, which was 28.43, 16.04, and 26.21% higher than T1, T2, and T4 treatments, respectively (*p* < 0.05). The trend of S-ALP activity was similar to S-SC activity, with the T3 treatment significantly increased by 15.30% compared to T1 (*p* < 0.05).

Moreover, as shown in Supplementary Table S1, SU-E and S-SC activities were significantly and positively correlated with TN content (*p* < 0.05), and highly significantly correlated with AN, AP, and AK content (*p* < 0.01). S-PPO activity was significantly and positively correlated with AK (*p* < 0.05), and S-CAT was significantly and positively correlated with pH and AK (*p* < 0.05). S-ALP activity was significantly and positively correlated with AN, and AP showed a highly significant correlation (*p* < 0.01) and significant correlation (*p* < 0.05) with AK.

### Sequencing analysis of microbial communities in Chinese chives rhizosphere soil

Based on high-throughput sequencing, 1,246,099 sequences of the bacterial community and 1,157,456 sequences of the fungal community were obtained from soil samples using B341F/B805R (bacterial 16SrRNA) and ITS3/ITS4 (fungal ITS) primer sets. Bacterial sequences ranged from 62,275–91,290 and fungal sequences from 73,849 to 75,571 for each treatment. The sequencing coverage was greater than 97% in all cases. The results were corroborated by the effective sequence length estimation of soil bacteria (Supplementary Figure S1) and dilution curve (Supplementary Figure S3) and the effective sequence length estimation of soil fungi (Supplementary Figure S2) and dilution curve (Supplementary Figure S4). This indicates that the sequencing results of this experiment can represent the actual situation of the samples and can reflect the structure of the soil microbial community.

**Figure 3:**
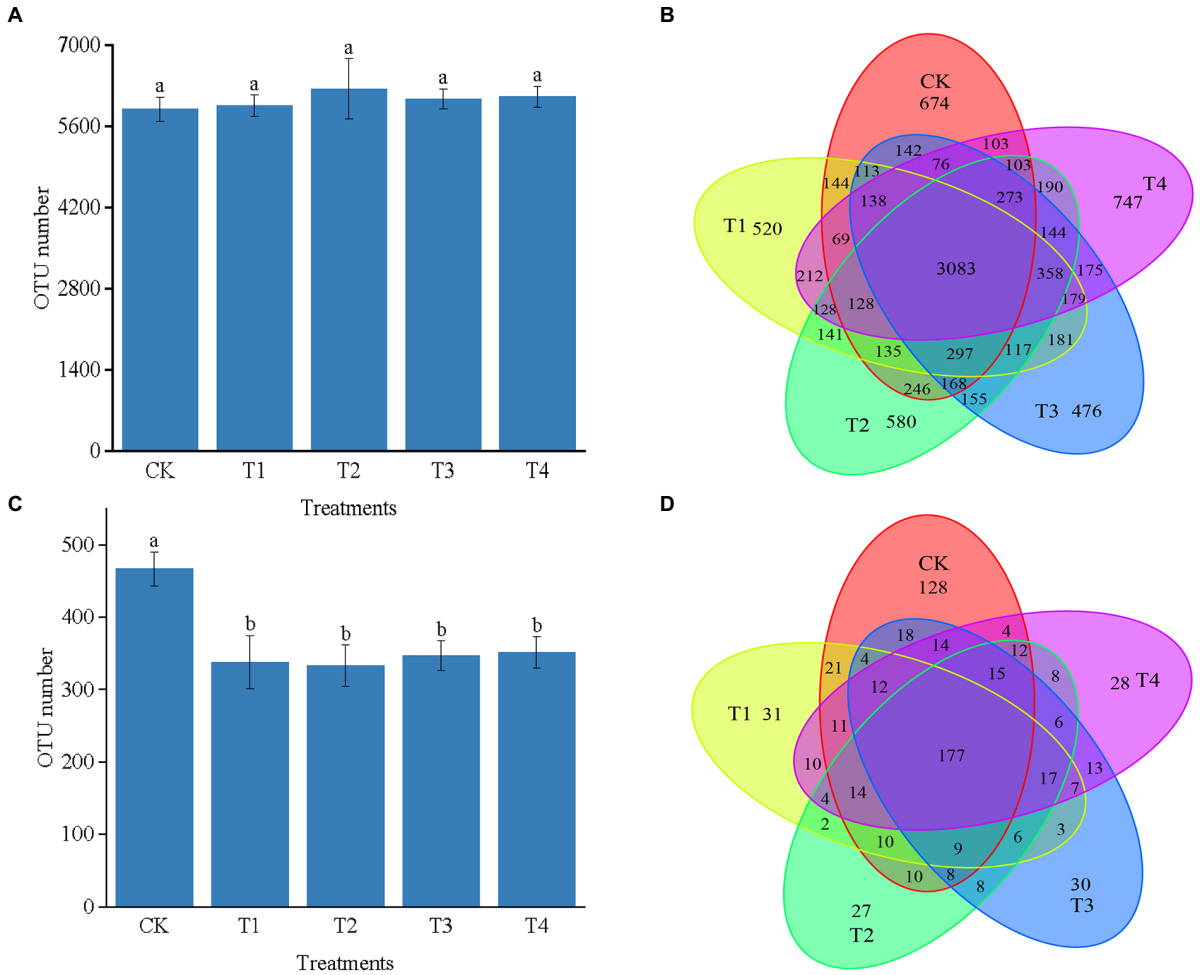
**(A)** The number of OTUs of bacterial in different treatments. **(B)** The Venn map for the characteristic number of Chinese chives rhizosphere bacterial under different fertilization treatments. **(C)** The number of OTUs of fungul in different treatments. **(D)** The Venn map for the characteristic number of Chinese chives rhizosphere fungul under different fertilization treatments. Values are the mean of three replicates. CK, no fertilizer application; T1, conventional fertilizer application; T2, reduced fertilizer application for slow-release fertilizer; T3, conventional fertilizer application for slow-release fertilizer; T4, reduced fertilizer application for conventional fertilizer. According to Duncan’s test, the average of the different letters (such as a, and b) in each column was significantly different at *p* < 0.05.

As shown in Figure 3A, the number of 16S rRNA OTUs in CK, T1, T2, T3, and T4 treatments were 5,829, 5,943, 6,246, 6,075, and 6,106, respectively. Number of bacterial sequences was the highest in T2 treatment Figure 3B indicates the differences in the characteristic number of rhizosphere microorganisms under different fertilization modes. The number of common characteristics among all the samples was 3,083. The unique features in CK, T1, T2, T3, and T4 treatments were 674, 520, 580, 476, and 747, respectively. Compared with CK, the number of bacterial sequence characteristics decreased by 154, 94, and 198 in the T1, T2, and T3 treatments, respectively, while it increased by 73 in the T4 treatment. The number of sequence characteristics was lower in the T1 and T3 treatments than in the T2 and T4 treatments, respectively, indicating that fertilizer application affected the number of soil bacterial sequence features.

The number of OTUs in the ITS could be divided into CK, T1, T2, T3, and T4 treatments with 467, 338, 333, 347, and 352, respectively. Further, the number of OTUs in each fertilization treatment was lower than that of CK, indicating that fertilization caused a decrease in the number of OTUs of soil fungi (Figure 3C). Figure 3D reveals the differences in the number of traits of fungal OTUs under different fertilization treatments. The number of common characteristics among all the samples was 177, and the number of features processed by CK, T1, T2, T3, and T4 was 128, 31, 27, 30, and 28, respectively. The number of unique fungal sequence features was 97, 101, 98, and 100 more in the CK treatment than in the T1, T2, T3, and T4 treatments. The number of features was higher in the T1 and T4 treatments than in the T3 and T2 treatments, respectively, indicating that fertilization reduced in the number of unique sequence features of the fungus in each treatment.

### Alpha diversity analysis of Chinese chives rhizosphere bacteria communities

As shown in Table 2, fertilizer application significantly affected soil bacteria and fungi diversity. The coverage values were all greater than 0.97, indicating that the number of sequence reads was sufficient to cover the microbial diversity in the soil. Compared with CK and T4, the soil bacterial alpha diversity index indicated, that the ACE index of T2 treatment increased by 12.81 and 10.61% (*p* < 0.05), respectively. The Chao1 index of T1, T2, and T3 treatments was higher than that of CK. The Shannon index of each treatment ranged from 7.20 to 7.45. Shannon index was the highest in CK and second highest in T2 treatment. Simpson’s index was 0.0015 to 0.0021 for each treatment, while the T2 treatment and CK had the closest Simpson’s index values. Soil fungal α-diversity indices showed that Chao1, Ace, Shannon, and Simpson indices were higher in the control treatment than in the fertilization treatment. This indicates that fertilization caused a decrease in the soil fungal α-diversity index.

**Table 2:** Alpha diversity analysis of Chinese chives rhizosphere microorganisms under different fertilization treatments.

	Treatments	Chao1	Ace	Shannon	Simpson	Coverage
Bacteria	CK	7252.30 ± 106.53ab	7362.36 ± 147.17b	7.45 ± 0.08a	0.0015 ± 0.0003b	0.9803 ± 0.0019a
	T1	7625.47 ± 743.35ab	7938.58 ± 894.14ab	7.20 ± 0.18ab	0.0021 ± 0.0006a	0.9767 ± 0.0027a
	T2	8050.91 ± 296.36a	8305.31 ± 351.33a	7.40 ± 0.05a	0.0015 ± 0.0001b	0.9756 ± 0.0013a
	T3	7671.26 ± 500.70ab	7892.62 ± 658.19ab	7.34 ± 0.03ab	0.0016 ± 0.0005ab	0.9772 ± 0.0024a
	T4	7283.49 ± 289.79ab	7508.95 ± 359.28b	7.30 ± 0.09ab	0.0019 ± 0.0003ab	0.9792 ± 0.0033a
Fungi	CK	570.08 ± 19.10a	565.24 ± 23.46a	4.29 ± 0.09a	0.0270 ± 0.0035b	0.9988 ± 0.0003a
	T1	425.69 ± 50.14b	422.23 ± 51.78b	3.88 ± 0.13a	0.0394 ± 0.0052b	0.9994 ± 0.0001a
	T2	420.31 ± 10.36b	413.10 ± 8.80b	3.97 ± 0.03a	0.0359 ± 0.0017b	0.9995 ± 0.0000a
	T3	439.45 ± 33.19b	435.35 ± 31.28b	3.59 ± 0.13ab	0.0591 ± 0.0067b	0.9991 ± 0.0003a
	T4	438.16 ± 38.20b	438.35 ± 38.46b	3.07 ± 0.49b	0.1829 ± 0.0120a	0.9987 ± 0.0007a

Chao1, ACE, Shannon, Simpson represent each index respectively; Values are reported as repeated mean ± standard error. According to Duncan’s test, the average of the different letters (such as a, b, and c) in each column was significantly different at p < 0.05. Coverage is the coverage rate of the sample library. CK, no fertilizer application; T1, conventional fertilizer application; T2, reduced fertilizer application for slow-release fertilizer; T3, conventional fertilizer application for slow-release fertilizer; T4, reduced fertilizer application for conventional fertilizer.

### Taxa number and distribution of Chinese chives rhizosphere microorganisms

**Figure 4:**
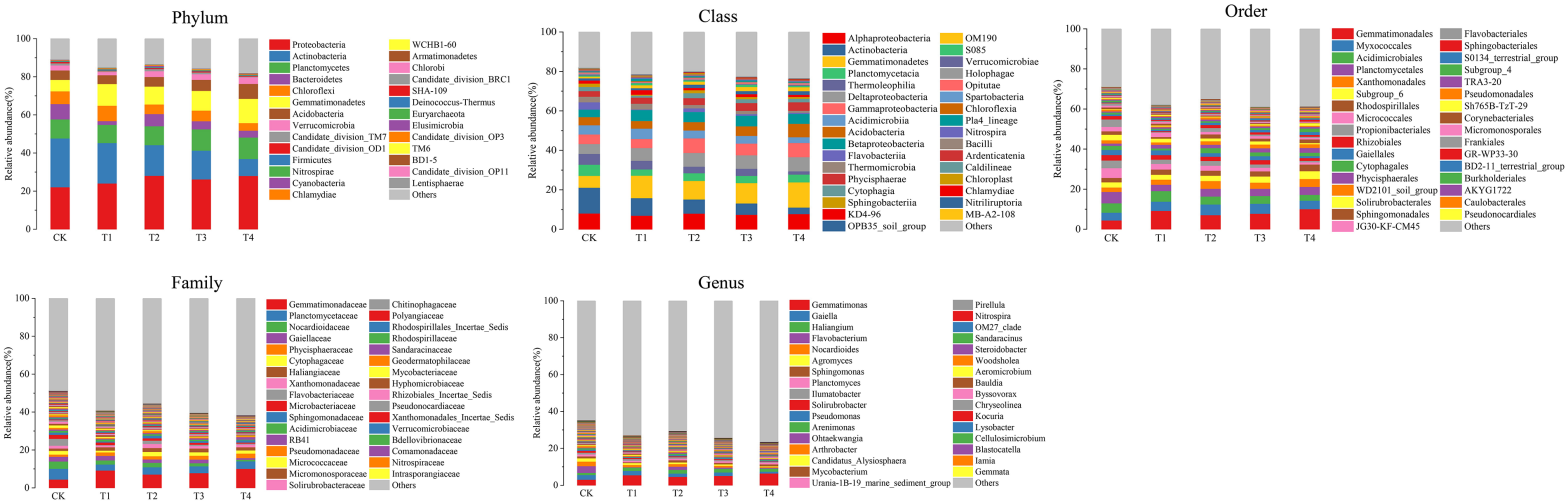
Species distribution at each level of Chinese chives rhizosphere bacterial under different fertilization treatments. Values are the mean of three replicates. CK, no fertilizer application; T1, conventional fertilizer application; T2, reduced fertilizer application for slow-release fertilizer; T3, conventional fertilizer application for slow-release fertilizer; T4, reduced fertilizer application for conventional fertilizer.

**Figure 5:**
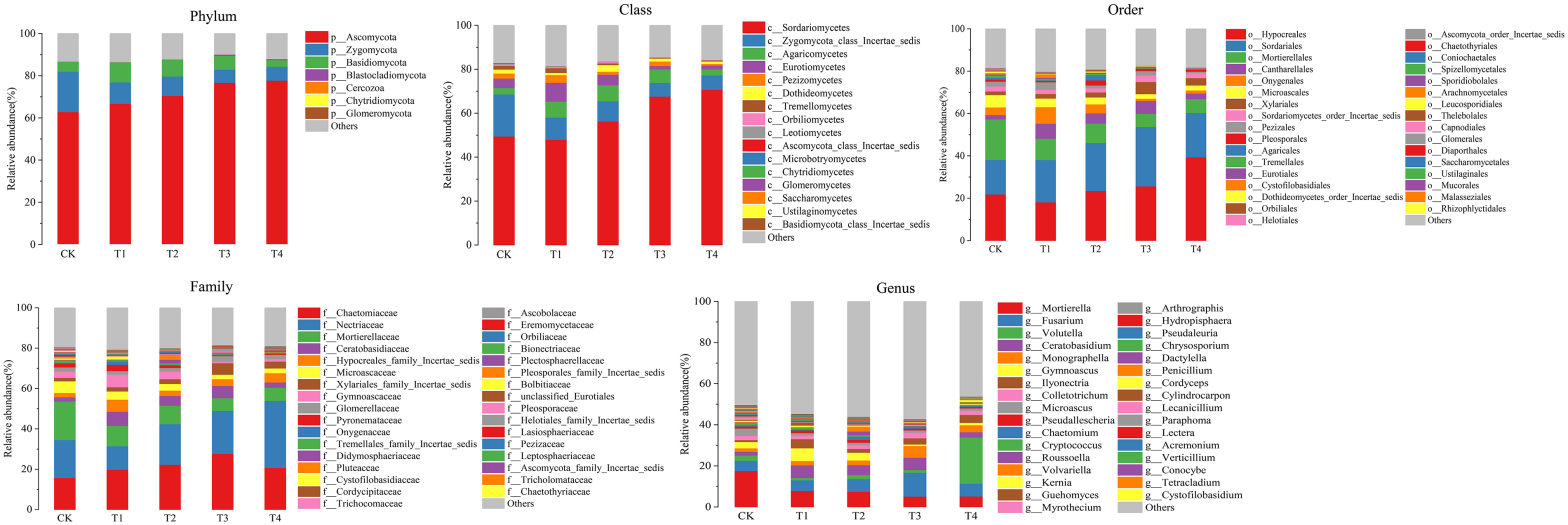
Species distribution at each level of Chinese chives rhizosphere fungul under different fertilization treatments. Values are the mean of three replicates. CK, no fertilizer application; T1, conventional fertilizer application; T2, reduced fertilizer application for slow-release fertilizer; T3, conventional fertilizer application for slow-release fertilizer; T4, reduced fertilizer application for conventional fertilizer.

**Figure 6:**
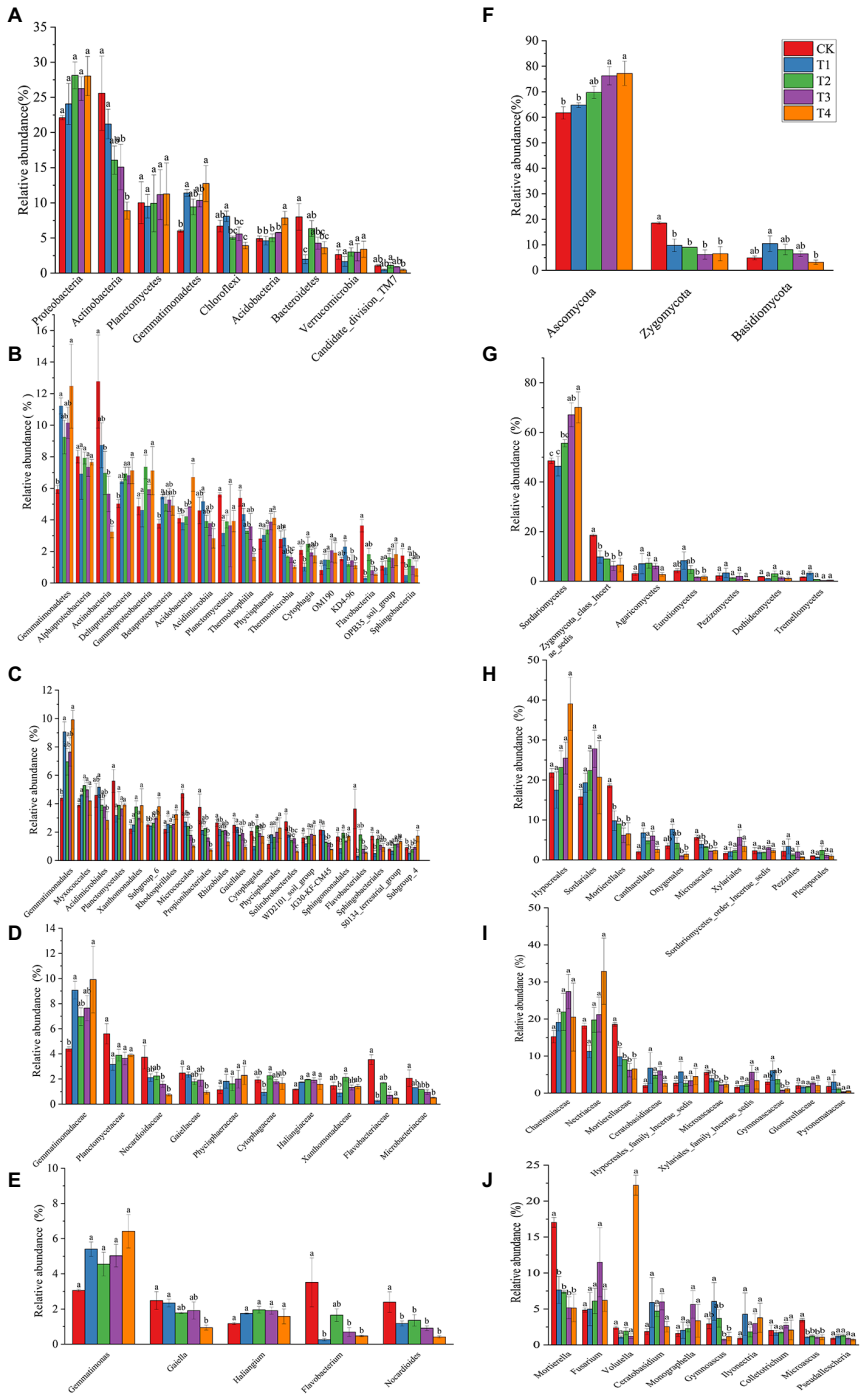
The relative abundance of the phyla for treatments under different fertilization treatments. **(A–E)** The bacterial with relative abundance > 1% in each treatment. **(F–J)** The fungal with relative abundance > 1% in each treatment. According to Duncan’s test, the different letters (such as a, b) on the graph bars represent significant difference at *p* < 0.05. CK, no fertilizer application; T1, conventional fertilizer application; T2, reduced fertilizer application for slow-release fertilizer; T3, conventional fertilizer application for slow-release fertilizer; T4, reduced fertilizer application for conventional fertilizer.

The number of species at each taxonomic level of rhizosphere microorganisms of Chinese chives under different fertilization treatments is shown in Table 3. Fertilization has resulted in changes in the number of bacterial and fungal species. The relative abundance of bacteria and fungi at each taxonomic level is shown in Figures 4, 5. At the bacterial phylum level, the dominant bacterial phyla (relative abundance >1%) were Actinobacteria, Proteobacteria, Planctomycetes, Bacteroidetes, Chloroflexi, Gemmatimonadetes, Acidobacteria Verrucomicrobia, and Candidate_division_TM7 (Figure 4 phylum). Compared to CK, the relative abundance of Gemmatimonadetes increased significantly (*p* < 0.05) in the T1 and T4 treatments, and the relative abundance of Actinobacteria, Chloroflexi, and Bacteroidetes relative abundance significantly decreased (Figure 6A; *p* < 0.05). However, Acidobacteria was significantly higher in the T4 treatment than in other treatments (*p* < 0.05). At the class level (Figure 4 Class), the relative abundance of Gemmatimonadetes, Deltaproteobacteria, and Betaproteobacteria increased in each treatment. The relative abundance of Gemmatimonadetes was significantly higher in the T1 and T4 treatments than in CK (*p* < 0.05). The relative abundance of Deltaproteobacteria was significantly higher (*p* < 0.05) and the relative abundance of Actinobacteria was significantly lower (*p* < 0.05) in the T2, T3, and T4 treatments compared to CK (Figure 6B). At the order level (Figure 4 Order), the relative abundance of *Gemmatimonadales*, *Myxococcales*, *Xanthomonadales*, and *Subgroup_6* increased, while the relative abundance of *Acidimicrobiales*, *Micrococcales*, *Propionibacteriales*, *Rhodospirillales*, *Rhizobiales*, *Gaiellales*, and *JG30-KF-CM45* decreased. The relative abundance of *Gemmatimonadales* was significantly higher in the T1 and T4 treatments than in CK (*p* < 0.05; Figure 6C). The relative abundance of Cytophagales and Sphingomonadales in the T2 treatment were significantly higher than those of T1 (*p* < 0.05; Figure 6C). At the family level (Figure 4 Family), *Gemmatimonadaceae*, *Phycisphaeraceae*, and *Haliangiaceae* increased in relative abundance. *Planctomycetaceae*, *Nocardioidaceae*, *Gaiellaceae*, *Flavobacteriaceae*, and the relative abundance of *Gemmatimonadaceae* was significantly higher (*p* < 0.05) in the T1 and T4 treatments compared to CK (Figure 6D). The relative abundance of *Cytophagaceae* and *Xanthomonadaceae* was significantly higher (*p* < 0.05) in the T2 treatment than in T1 (Figure 6D). At the genus level (Figure 4 Genus), relative abundance of *Gemmatimonas*, *Haliangium* increased and that of *Gaiella*, *Flavobacterium*, *Nocardioides*, and *Agromyces* decreased. The relative abundance of *Gaiella* was significantly higher (*p* < 0.05) in the T1 treatment than in T4 (Figure 6E). The relative abundance of *Flavobacterium* was significantly lower in the T1, T3, and T4 treatments compared to CK, and the relative abundance of *Nocardioides* was significantly lower (*p* < 0.05) in T1, T2, T3, and T4 treatments (Figure 6E).

**Table 3:** The number of species at each level of Chinese chive rhizosphere bacterial under different fertilization treatments.

	Treatment	Phylum	Class	Order	Family	Genus
Bacteria	CK	27.00 ± 0.33a	48.00 ± 0.68a	90.00 ± 0.33a	148.00 ± 0.33a	252.00 ± 3.53a
	T1	27.00 ± 0.33a	48.00 ± 0.57a	89.00 ± 2.73a	143.00 ± 3.84a	237.00 ± 11.35a
	T2	27.00 ± 0.58a	50.00 ± 1.20a	92.00 ± 1.45a	149.00 ± 2.31a	252.00 ± 10.12a
	T3	27.00 ± 0.58a	49.00 ± 0.88a	90.00 ± 0.33a	140.00 ± 1.33a	230.00 ± 1.15a
	T4	27.00 ± 0.58a	49.00 ± 1.76a	90.00 ± 1.76a	141.00 ± 3.79a	234.00 ± 10.12a
Fungi	CK	7.00 ± 0.00a	15.00 ± 0.33a	34.00 ± 0.33a	61.00 ± 2.00a	93.00 ± 5.21a
	T1	6.00 ± 0.33a	15.00 ± 1.20a	31.00 ± 1.53ab	53.00 ± 3.93ab	72.00 ± 6.44b
	T2	7.00 ± 0.33a	15.00 ± 1.33a	34.00 ± 1.00a	55.00 ± 1.73ab	78.00 ± 3.53ab
	T3	7.00 ± 0.33a	14.00 ± 1.45a	31.00 ± 1.76ab	53.00 ± 1.53ab	74.00 ± 4.84ab
	T4	7.00 ± 0.33a	14.00 ± 0.67a	28.00 ± 1.33b	51.00 ± 3.06b	79.00 ± 5.03ab

Unit, piece; values are the mean of three replicates. CK, no fertilizer application; T1, conventional fertilizer application; T2, reduced fertilizer application for slow-release fertilizer; T3, conventional fertilizer application for slow-release fertilizer; T4, reduced fertilizer application for conventional fertilizer. Values are reported as repeated mean ± standard error. According to Duncan’s test, the average of the different letters (such as a, b, and c) in each column was significantly different at P < 0.05.

At the fungal phylum level (Figure 5 Phylum), the relative abundance of Ascomycota, Zygomycota, and Basidiomycota were all greater than 1%, and that of Ascomycota in each treatment exceeded 70%. Ascomycota’s relative abundance in the T3 treatment was significantly higher than that in T1, and Basidiomycota’s relative abundance in the T1 treatment was significantly higher than that in T4 (*p* < 0.05; Figure 6F). The relative abundance of Zygomycota was significantly lower (*p* < 0.05) in each fertilization treatment compared to CK (Figure 6F). At the class level (Figure 5 Class), the relative abundance of Sordariomycetes and Agaricomycetes increased, and relative abundance of Zygomycota_class_Incertae_sedis and Eurotiomycetes decreased. Compared with CK, the relative abundance of Sordariomycetes increased significantly (*p* < 0.05) in the T3 and T4 treatments.The relative abundance of Eurotiomycetes was significantly higher (*p* < 0.05) in T1 treatment than in T3, but the relative abundance of Sordariomycetes was significantly higher (*p* < 0.05) in the T3 treatment than in T1 (Figure 6G). At the order level (Figure 5 Order), the relative abundance of *Hypocreales*, *Sordariales*, *Cantharellales*, and *Xylariales* increased, and the relative abundance of *Mortierellales*, *Onygenales*, *Microascales*, and *Pezizales* decreased in the T1, T2, T3, and T4 treatments. The relative abundance of *Mortierellales* decreased significantly (*p* < 0.05) compared to CK, and the relative abundance of *Onygenales* increased significantly (*p* < 0.05) in the T1 treatment compared to the T3 and T4 treatments (Figure 6H). At the family level (Figure 5 Family), the relative abundance of *Chaetomiaceae*, *Nectriaceae*, *Ceratobasidiaceae*, *Hypocreales_family_Incertae_sedis*, and *Xylariales_family_Incertae_sedis* increased and the relative abundance of *Mortierellaceae*, *Microascaceae*, *Gymnoascaceae*, and *Pyronemataceae* decreased. The relative abundance of *Mortierellaceae* decreased significantly (*p* < 0.05) in the T1, T2, T3, and T4 treatments compared to CK. The relative abundance of *Gymnoascaceae* was significantly higher (*p* < 0.05) in the T1 treatment than in T3 and T4 (Figure 6I). At the genus level (Figure 5 Genus), the relative abundance of *Mortierella*, *Microascus*, and *Gymnoascus* decreased, and that of *Fusarium*, *Ceratobasidium*, *Monographella* and *Ilyonectria* increased. As shown in Figure 6J, the relative abundance of *Mortierella* and *Microascus* decreased significantly (*p* < 0.05) in each fertilization treatment compared to CK. The relative abundance of *Gymnoascus* decreased significantly (*p* < 0.05) in the T3 and T4 treatments compared to T1. The relative abundance of *Volutella* increased significantly in the T4 treatment.

### Analysis of PCoA among different fertilization treatments

**Figure 7:**
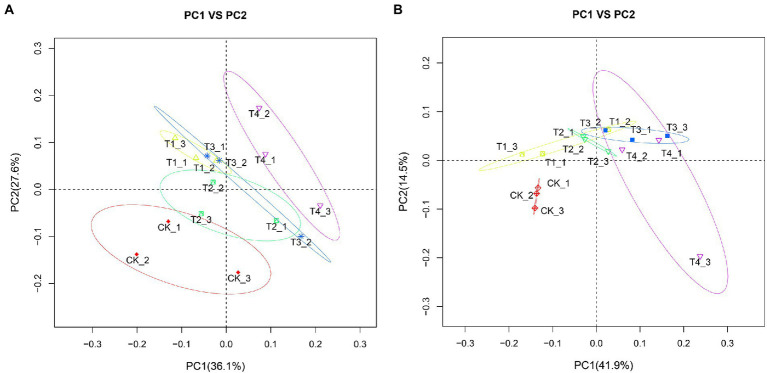
Principal coordination analysis (PCoA) plots with weighted UniFac distance metric in soil samples under different fertilization treatments. **(A)** Bacterial PCoA plot. **(B)** Fungal PCoA plot. CK, no fertilizer application; T1, conventional fertilizer application; T2, reduced fertilizer application for slow-release fertilizer; T3, conventional fertilizer application for slow-release fertilizer; T4, reduced fertilizer application for conventional fertilizer.

To clarify the changes in soil community structure between the rhizosphere of each fertilization treatment and the control, we calculated the β-diversity index of soil bacteria and fungi communities (Figure 7). After analyzing the soil bacterial PCoA data (Figure 7A), PCoA1 and PCoA2 explained 36.1 and 27.6% of the differences in soil bacterial communities, respectively. Further, there were significant differences between the fertilization treatments compared with CK. The distance between treatments T1 and T4 was also farther, indicating that the difference in bacterial flora between them was significant, suggesting that the amount of fertilizer applied might affect the flora. The distance between treatments T2 and T4 was farther. However, the amount of fertilizer applied to both was the same, suggesting that the type of fertilizer applied also affected the flora under reduced fertilizer application. After analyzing the soil fungi PCoA data (Figure 7B), PCoA1 and PCoA2 explained 41.9 and 14.5% of the differences in soil bacterial communities, respectively. The three repeats of CK were clustered together, showing good repeatability. We recorded that the distance between treatments T2 and T4 was relatively close; however, there was no overlapping between treatments, suggesting that the type of fertilizer applied still affected the fungal flora in the case of reduced fertilization. Separation was also observed between the T2 and T3 treatments, indicating that the amount of fertilizer applied also affects the fungal flora when the same slow-release fertilizer is applied.

### Analysis of RDA among different fertilization treatments

**Figure 8:**
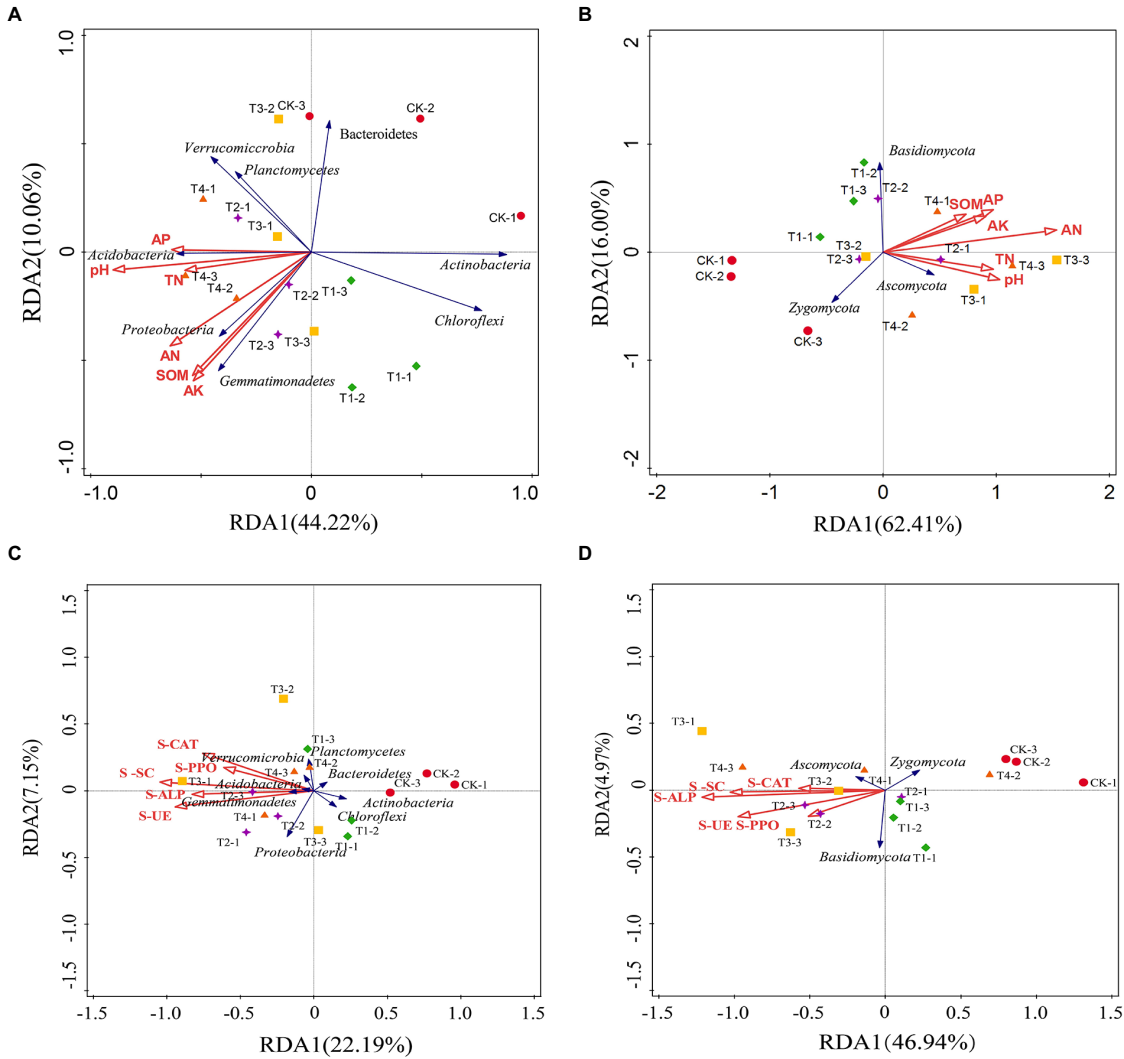
Redundancy analysis (RDA) of the abundant phyla and soil samples from different fertilization treatments. **(A,B)** The relationship between the soil bacterial taxa and the soil properties and enzyme activities. **(C,D)** The relationship between the soil fungal taxa and the soil properties and enzyme activities. SOM, soil organic matter; TN, total nitrogen; AN, alkali-hydrolyzable nitrogen; AP, available phosphorus; AK, available potassium; S-UE, soil urease; S-PPO, soil polyphenol oxidase; S-CAT, soil catalase; S-SC, soil sucrase; S-ALP, soil alkaline phosphatase. CK, no fertilizer application; T1, conventional fertilizer application; T2, reduced fertilizer application for slow-release fertilizer; T3, conventional fertilizer application for slow-release fertilizer; T4, reduced fertilizer application for conventional fertilizer.

Redundancy analysis was used to analyze the relationship between the structure (relative abundance) of eight bacterial communities and three fungal communities with relative abundance > 1% and the physical and chemical properties of the soil and soil enzyme activity (Figure 8). In the redundancy analysis of bacterial community structure and soil physicochemical properties (Figure 8A), the first and second ordination axes clarified 44.22 and 10.06% of the changes in the soil bacterial community, respectively, explaining 54.28% of the total variation of the soil bacterial community. The explanation for the variation of the soil microbial community was evident. Proteobacteria and Gemmatimonadetes were positively correlated with soil AN, SOM, and AK, while Acidobacteria, Actinobacteria, and Chloroflexi were negatively correlated with soil pH, TN, AN, AP, AK, and SOM. Acidobacteria were positively correlated with soil pH, TN, and AP, suggesting that soil physicochemical properties can affect soil bacteria. Three fungal phyla and soil physicochemical properties with relative abundance greater than 1% were selected for RDA analysis (Figure 8B). The first and second axes explained 78.41% of the total variation in the soil fungal community. Ascomycota was positively correlated with soil TN, SOM, AK, AP, and pH, while Zygomycota was the opposite.

In the redundancy analysis of bacterial community structure and soil enzyme activity (Figure 8C), the first and second ordination axes explained 22.19 and 7.15% of the variation in the soil fungal community and 29.34% of the total variation in the soil fungal community, respectively. Proteobacteria, Gemmatimonadetes, Acidobacteria, and Verrucomicrobia were positively correlated with SU-E, S-ALP, S-SC, S-PPO, and S-CAT, while Bacteroidetes, Actinobacteria, and Chloroflexi were negatively correlated. In the redundancy analysis of soil fungal community structure and soil enzyme activity (Figure 8D), the first and second ordination axes together explained 71.91% of the degree of variation. Ascomycota was positively correlated with SU-E, S-ALP, S-SC, S-PPO, and S-CAT, and Zygomycota was negatively correlated. Basidiomycota was positively correlated with SU-E and S-PPO.

## Discussion

### Response of soil properties and enzyme activities to different fertilization treatments

Soil physicochemical properties result from the combined action of the soil, plants, and the surrounding environment. They are the essential characterization of soil quality change and development and are the core content of this study (Manrubia et al., 2019). Available soil nutrient content directly determines the size of soil nutrient supply capacity (Jing et al., 2020). Studies have shown that owing to the slow-release effect, slow release fertilizers can be released slowly over a longer period of time after application into the soil, keeping the nitrogen and phosphorus content of the soil increased and at a high level (Wang et al., 2019), which was also mentioned in this trial. In this study, the TN, AN, and AP contents of treatments T2 and T3 with slow-release fertilizer application were higher than the other treatments (Figure 1). This indicates that the application of slow-release fertilizer can evidently enhance soil TN, AN, and AP contents and increase soil nutrient content compared to common fertilizer. Similarly, the soil nutrient content of the reduced fertilizer treatments T2 and T4 was higher than that of the regular fertilizer treatments T1 and T3. The AK content of the T4 treatment with additional potassium fertilizer was not significantly higher than that of the T1 treatment, indicating that over-fertilization did not increase the soil nutrient content (Ding et al., 2020). Fertilizer application caused a change in soil C/N ratio, an increase in soil nitrogen content, and a decrease in soil organic matter content (Li et al., 2019). The high level of TN and low SOM in the soils of T2 and T3 treatments in this experiment satisfactorily substantiated this view. Furthermore, in the previous study on yield, Chinese chives yield was significantly increased in T2 and T3 treatments (Niu et al., 2020), indicating that its vigorous growth and root distribution, and infiltration in the soil also changed the soil physical and chemical properties and further increased soil nutrient content (Li et al., 2016).

Soil enzyme activity affects soil nutrient cycling, and the activity is influenced by the rate and type of fertilization applied (Teng et al., 2018; Fertahi et al., 2021). Gong et al. (2019) showed that soil enzyme activity was positively correlated with soil nutrient content, indicating that the higher the soil enzyme activity, the more soil nutrient content was accumulated. The soil enzymes selected in this experiment (urease, sucrase, alkaline phosphatase, and catalase) are involved in the soil C, N, and P cycles (Attademo et al., 2021). In the present experiment, compared with conventional fertilizer application, slow-release fertilizer application was able to increase soil enzyme activities, including SU-E, S-SC, and S-ALP. Meanwhile, the comparison of soil enzyme activities in T2 and T3 treatments showed that soil enzyme activities did not change with the amount of fertilizer applied (Teng et al., 2018; Liu et al., 2021). The main role of S-PPO was to break down harmful substances in root secretions and reduce the self-toxicity of plants (Jian et al., 2016). The vigorous growth of Chinese chives after fertilization causes the accumulation of harmful substances in the root secretions, which indirectly increases the number and activity of secretion of S-PPO by soil microorganisms (Qu et al., 2022). S-CAT activity was closely related to soil organic matter content (Jian et al., 2016). The T2 treatment had the lowest organic matter content; however, the S-CAT activity was significantly higher than the other treatments (*p* < 0.05), corroborating the above conclusion. After the soil organic matter content decreased, the phosphatase activity increased, similar to the previous study result (Jing et al., 2020).

### Response of soil microbial diversity to different fertilization treatments

The slow-release fertilizers also affects soil physical and chemical properties nutrient content, affecting soil microbial community diversity (Tian et al., 2019; Chen et al., 2022). The Chao1, Ace, Shannon, and Simpson indices of soil bacteria increased under different fertilization treatments (Table 2), especially in the T2 treatment. The Ace index of T2 treatment was significantly higher than that of CK, and the Shannon index decreased the least. Meanwhile, Chao1 and Ace indices of soil bacteria increased with increasing fertilizer application whereas the Shannon and Simpson indices decreased with increasing fertilizer application when common fertilizer was applied, similar to the findings of Rughoft et al. (2016). However, the changes in soil bacterial diversity indices of slow-release fertilizer treatments were exactly the opposite, indicating that excessive application of slow-release fertilizer did not increase soil bacterial diversity. The diversity indices of soil fungi were all decreased compared to CK, the T2 treatment had the smallest decrease in Shannon and increase in Simpson index. Similarly, the soil fungal diversity index decreased after fertilization, especially in the T2 and T3 treatments, which indirectly indicated that the application of slow-release fertilizer had a positive effect on the control of soil-borne diseases and increased the uptake of soil nutrients by roots (Jarvis et al., 2015). Similarly, soil nutrients were increased after fertilizer application and soil physical and chemical properties were changed. The slow-release properties of slow-release fertilizers, on the other hand, allow the nutrients in the soil to be maintained at a high level for a certain period of time (Chen et al., 2022). This allows the proliferation of soil bacteria to become more stable and inhibit the soil fungi, which account for a small percentage of the total. This in turn leads to an increasing bacterial diversity and decreasing fungal diversity (Hu et al., 2017; Gong et al., 2019). Therefore, applying slow-release fertilizers can facilitate sustainable agricultural development in terms of changes in the abundance and diversity of soil microorganisms.

In the PCoA analysis, the bacterial and fungal communities of each fertilization treatment produced separation from CK. This indicates that fertilizer application can affect soil microbial community structure by changing soil nutrient content (Liang et al., 2020). Areas between T1 and T4 treatments did not overlap, but areas between T2 and T3 treatments did, suggesting that changes in microbial community structure in the common fertilizer treatment may be related to the amount of fertilizer applied. Similarly, in the PCoA analysis of soil bacteria, the T2, T3, and T1 treatments overlapped concertedly, but the T4 treatment was separated from these three treatments, which may be related to the lower total fertilizer application in the T4 treatment. In contrast, there was a crossover between all fertilization treatments for fungi, reflecting that bacteria are more sensitive to fertilization than fungi (Gong et al., 2019).

### Response of soil microbial community structure to different fertilization treatments

Changes in the composition of soil microbial communities affect soil nutrient changes to some extent (Teng et al., 2018). In this study, the differences in the relative abundance of soil bacteria between treatments (T2 and T3) with slow-release fertilizer application were small. This indicates that slow-release fertilizers have less effect on soil bacterial communities. In contrast, significant differences in the relative abundance of bacteria were observed between treatments (T1 and T4) that applied common chemical fertilizers, indicating that the amount of common chemical fertilizers applied can affect the bacterial community. The relative abundance of Proteobacteria, Gemmatimonadetes, and Acidobacteria was increased, and Actinobacteria, Chloroflexi, and Bacteroidetes were decreased (Figures 4, 6A). Proteobacteria contain certain subclasses of bacteria that can fix atmospheric nitrogen (Li et al., 2019). The increase in soil nitrogen content after fertilization stimulated the rapid proliferation of Proteobacteria, and the nitrogen-fixing bacteria in Proteobacteria became more active and increased their nitrogen fixation capacity, resulting in nitrogen accumulation. After nitrogen accumulation, Proteobacteria proliferated, and their relative abundance increased (Sabir et al., 2021). The overwintering production of Chinese chives is mainly conducted in greenhouses, which are closed for a long-time during production and have low gas exchange with the external environment, leading to a relative decrease in oxygen content in the greenhouse. The relative abundance of Actinobacteria in T4, the least fertilized treatment, decreased significantly (*p* < 0.05) compared with T1, indicating that the changes in the relative abundance of Actinobacteria were affected not only by soil oxygen content but also by the amount of fertilizer applied (Figure 6A; Li et al., 2021). The relative abundance of parthenogenic anaerobic Gemmatimonadetes was increased, and aerobic Actinobacteria and Chloroflexi growth were suppressed (Figures 4, 6A; Zhao et al., 2016). Chloroflexi relative abundance was significantly higher in the T1 treatment than in the T4 treatment (*p* < 0.05), but there was no significant difference between T2 and T3. This suggests that Chloroflexi may be more sensitive to changes in fertilizer application of common chemical fertilizers. The class of Gemmatimonadetes was vulnerable to soil pH, and its relative abundance was higher in alkaline soil (Figures 4, 6B). It was especially the T4 treatment that had the highest pH and the highest relative abundane of Gemmatimonadetes (Figures 1, 6B), that result was consistent with the studies of Zhang et al. (2017). The relative abundance of *Acidimicrobiales*, an oligotrophic bacterium, was inversely correlated with soil organic matter content (Zhou et al., 2015). This was corroborated by the decrease in the relative abundance of *Acidimicrobiales* in this study (*p* < 0.05, Figure 6C). *Nocardioidaceae* and *Microbacteriaceae* also participate in the carbon and nitrogen cycle in the soil. However, when the N content in the soil is too high or too low, it affects their growth and development, resulting in a decrease in their relative abundance (Figure 6D; Chen et al., 2022). *Nocardioides*, as aerobic bacteria, were one of the Actinobacteria (Zhao et al., 2016). It also participates in the soil nitrogen cycle and its relative abundance decreases (*p* < 0.05,Figure 6E), partly owing to a decrease in the oxygen content of the soil and partly owing to a change in the soil C/N ratio and inhibition of growth and development by other genera with higher relative abundance (Jiao et al., 2016).

Unlike the bacterial community, the relative abundance of fungi increased in each treatment, mainly owing to the increase in soil nutrients after fertilization, especially phosphorus, leading the rapid proliferation of fungi and more nutrients from decomposing organic matter, thereby promoting the relative abundance of fungi (Wang et al., 2018; Ye et al., 2020; Wu L. et al., 2021). However, as with bacteria, the fungal community was also sensitive to changes in the amount of common chemical fertilizers applied. For example, the relative abundance of Ascomycota was significantly higher in the T4 treatment than in the T1 treatment. In the overwintering production of Chinese chives, the environment in the greenhouse was suitable for the rapid growth of Chinese chives. The root secretions of Chinese chives are abundant at this time. The various sugars, amino acids, and organic acids in root secretions can also promote the growth of ascomycetes and other heterotrophic fungi (Abiraami et al., 2020; Gao et al., 2021). Compared with no fertilization, the relative abundance of Ascomycota in the soil treated with fertilization was higher (*p* < 0.05, Figure 5). The relative abundance of Ascomycota in each treatment exceeded 60% (Figure 6F), followed by Zygomycota and Basidiomycota, consistent with the conclusion of earlier studies (Li and Wu, 2018). The relative abundance of Zygomycota, a saprophytic fungus, increased with the availability of nutrients provided by fertilization treatments (Richardson, 2009). In this study, the relative abundance of Zygomycota decreased in the T3 and T4 treatments, indicating that nitrogen application above or below a certain threshold can change the soil C/N ratio and affect the fungal community structure (Wang et al., 2017). The relative abundance of the T4 treatment in the Basidiomycota decreased mainly because most of the fungi in Basidiomycota are symbiotic with plants and rely on plant-synthesized carbon to survive (Nie et al., 2018). The T4 treatment applied the least amount of fertilizer, and the chives plants were weakly grown and could not be provided with enough nutrients to supply Basidiomycota development. At the class level, the relative abundance of Sordariomycetes in all treatments exceeded 45% (Figure 6G) and was the dominant class, consistent with the findings of other studies (Zhou et al., 2016). After fertilization, the relative abundance of Sordariomycetes was lower in the T1 treatment than in the T3 and T4 treatments, indicating that Sordariomycetes were less affected by the type and amount of fertilizer applied when soil nutrients were sufficient (Ding et al., 2017). Eurotiomycetes lead to soil nitrogen loss and greenhouse gas production (Mothapo et al., 2015). In contrast, the relative abundance of Eurotiomycetes decreased after the slow-release and reduced fertilizer applications (*p* < 0.05), which laterally indicated that the slow-release and reduced fertilizer applications could reduce soil nitrogen loss. The relative abundance of *Mortierellales* and *Microascales*, as pathogenic fungi, decreased after the slow-release fertilizer application (*p* < 0.05, Figure 6H), indicating that the application of slow-release fertilizer could reduce the chance of soil-borne diseases (Ding et al., 2017).

The abundance of soil microorganisms is closely related to the soil environment, and the physical and chemical properties of the soil affect the abundance and activity of microorganisms (Cruz-Martinez et al., 2012). In this study, the slow-release fertilizer facilitated the improvement of soil physicochemical environment (Figures 1, 2). RDA analysis showed that soil pH, TN, AN, AP, AK, and SOM were positively correlated with Proteobacteria (Grieneisen et al., 2019), and negatively correlated with Actinobacteria, Bacteroidetes, and Chloroflexi (Figure 8A; Ge et al., 2008). Proteobacteria were positively correlated with TN and AN, which were related to their inclusion of nitrogen-fixing bacteria (Moulin et al., 2001). Nitrogen-fixing bacteria fix and release nitrogen from the air into the soil, increasing in soil N contentand promoting the proliferation of Proteobacteria. Actinobacteria were negatively correlated with soil pH, consistent with the conclusion (Cederlund et al., 2014). Verrucomicrobia was an oligotrophic bacterium and the various types of nutrients in the soil had little effect on its activity and relative abundance (Zhou et al., 2015), which was also verified by RDA analysis. Chloroflexi was negatively correlated with TN, which is similar to the findings of Zhalnina et al. (2015). Lauber et al. (2009) and Rousk et al. (2010) showed that the abundance of most phyla was pH dependent and the relative abundance of Proteobacteria, Gemmatimonadetes, Verrucomicrobia, and phytoplankton were positively related to soil pH and they indicated that soil pH was an important factor affecting soil microorganisms. RDA analysis also showed that soil enzyme activity also affected soil bacterial community structure. Soil enzyme activity was positively correlated with Proteobacteria, Planctomycetes, Gemmatimonadetes, Verrucomicrobia, and Acidobacteria and negatively correlated with Actinobacteria, Chloroflexi, Bacteroidetes (Figure 8C), similar to study findings (Liu et al., 2021). The negative correlation between Actinobacteria and soil enzyme activity is that Actinobacteria were aerobic bacteria (Gong et al., 2019), and the increase in soil enzyme activity was more favorable for soil nutrient transformation, and roots and other microorganisms were more active and consumed more oxygen. Actinobacteria activity was inhibited when the oxygen content in the soil decreased. The above results indicate that the effects of different fertilization treatments on soil bacterial communities were mainly achieved by changing soil physical and chemical properties and enzyme activities.

Ascomycota showed a positive correlation with soil pH, TN, AN, AP, AK, and SOM (Figure 8B), similar to the results of Wu X. et al. (2021). Ascomycota plays a major role in the decomposition of organic matter in the soil, and soil nutrient changes in the soil certainly have an impact (Fuke et al., 2021). Furthermore, Ascomycota has been shown to promote nitrogen accumulation, and its community structure and nitrogen changes were significantly correlated, consistent with our study results (Wu B. B. et al., 2021). On the contrary, Zygomycota was negatively correlated with soil physicochemical properties, probably owing to competition among microorganisms for soil nutrients, resulting in the inhibition of normal growth of Zygomycota (Nie et al., 2018). Similarly, RDA analysis of soil fungi and enzyme activities showed that SU-E, S-ALP, S-CAT, S-PPO, and S-SC were positively correlated with Ascomycota and negatively correlated with Zygomycota (Figure 8D). This result could be attributed to the fact that soil fungi tended to be heterotrophic fungi and Ascomycota was the dominant phylum, which utilized soil nutrients to a significantly higher extent than other fungi ，and also formed an inhibitory effect on other fungi (Nie et al., 2018; Wu X. et al., 2021). In conclusion, the alteration of soil fungal communities also occurred due to different fertilization treatments that changed soil physicochemical properties and enzyme activities.

## Conclusion

Fertilizer application affected soil physicochemical properties, microbial diversity index, and community structure, indicating that soil microorganisms responded differently to fertilizer application amount and type. Compared with conventional fertilizer application (common chemical fertilizer), slow-release fertilizer application significantly enhanced total soil nitrogen and alkaline soluble nitrogen, soil urease, and sucrase activities. It facilitated the conversion of soil organic matter, and changes the soil nutrient content, thus affecting the soil microbial community structure and diversity. Increased the relative abundance of Proteobacteria and Ascomycota, decreased the relative abundance of Actinomycota, Chloroflexi, and Basidiomycota, and reduced the fertilizer input. Furthermore, the reduced fertilizer application treatment (T2: N 29.2 kg·667 m^−2^, P_2_O_5_ 12.0 kg·667 m^−2^, K_2_O 21.6 kg·667 m^−2^) of slow-release fertilizer was better, which could reduce fertilizer application, reduce fund investment, and increase economic interests.

## Data availability statement

The datasets presented in this study can be found in online repositories. The names of the repository/repositories and accession number(s) can be found at: https://www.ncbi.nlm.nih.gov/, PRJNA852613 and PRJNA852643.

## Author contributions

JX, JL, JZ, and XZ designed the experiments. TN performed the experiments, analyzed the data, and wrote the manuscript. JX, JL, JZ, and CW critically revised the manuscript. HM organized the pictures and tables. All authors read and approved the final manuscript.

## Funding

This work was supported by the National Key Research and Development Program of China (2016YFD0201005); the Special Fund for Technical System of Melon and Vegetable Industry of Gansu Province (GARS-GC-1), China.

## Conflict of interest

HM was employed by Lanzhou New Area Agricultural Science and Technology Development Co., Ltd.

The remaining authors declare that the research was conducted in the absence of any commercial or financial relationships that could be construed as a potential conflict of interest.

The reviewer YW declared a shared affiliation with the authors TN, JX, JL, JZ, XZ, and CW to the handling editor at the time of review.

## Publisher’s note

All claims expressed in this article are solely those of the authors and do not necessarily represent those of their affiliated organizations, or those of the publisher, the editors and the reviewers. Any product that may be evaluated in this article, or claim that may be made by its manufacturer, is not guaranteed or endorsed by the publisher.

## Footnotes

^1^ http://www.R-project.org/
