# Supplementary material for: Response of rhizosphere microbial community of Chinese chives under different fertilization treatments
Source: Front Microbiol. 2022 Nov 21;13:1031624. doi: 10.3389/fmicb.2022.1031624 (PMC9719922; doi:10.3389/fmicb.2022.1031624)
Supplement: Supplementary file 1 [file Data_Sheet_1.docx]

**TABLE.S1 Correlation coefficients of soil enzymes and soil physical and chemical properties under different fertilization treatments**

| Correlation | S-UE | S-PPO | S-CAT | S-SC | S-ALP |
| --- | --- | --- | --- | --- | --- |
| pH | 0.504 | 0.272 | 0.627* | 0.554* | 0.297 |
| TN | 0.591* | 0.424 | 0.475 | 0.62* | 0.385 |
| AN | 0.862** | 0.491 | 0.389 | 0.793** | 0.858** |
| AP | 0.714** | 0.48 | 0.502 | 0.715** | 0.667** |
| AK | 0.892** | 0.607* | 0.567* | 0.714** | 0.571* |
| SOM | 0.286 | 0.082 | 0.212 | 0.164 | 0.158 |

Note: * P ＜0.05; ** P ＜0.01. TN is total soil nitrogen; AN is alkali-N; AP is available phosphorus; AK is available potassium; SOM is soil organic matter.


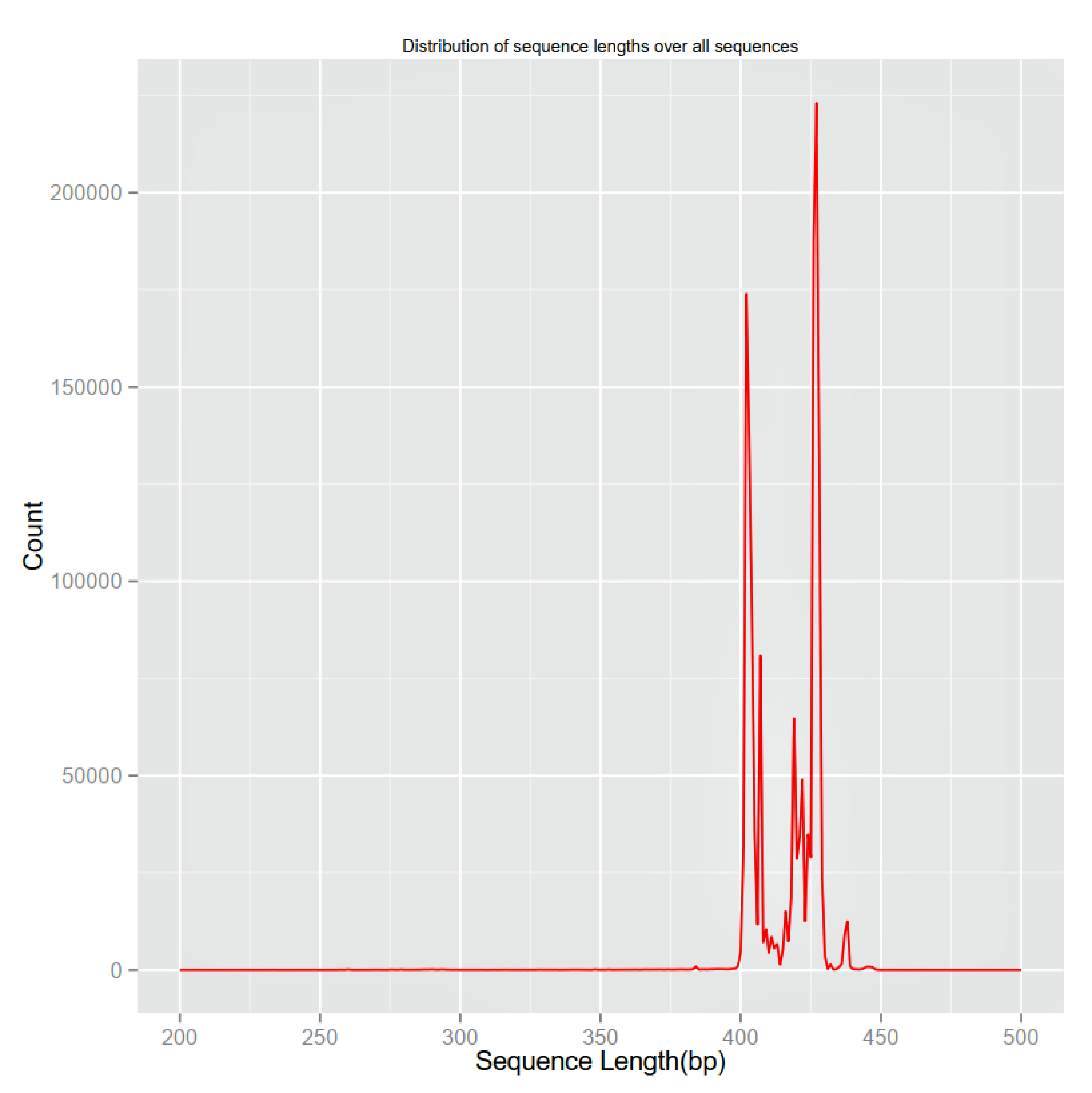


**FIGURE.S1 Soil bacteria effective sequence length estimation**


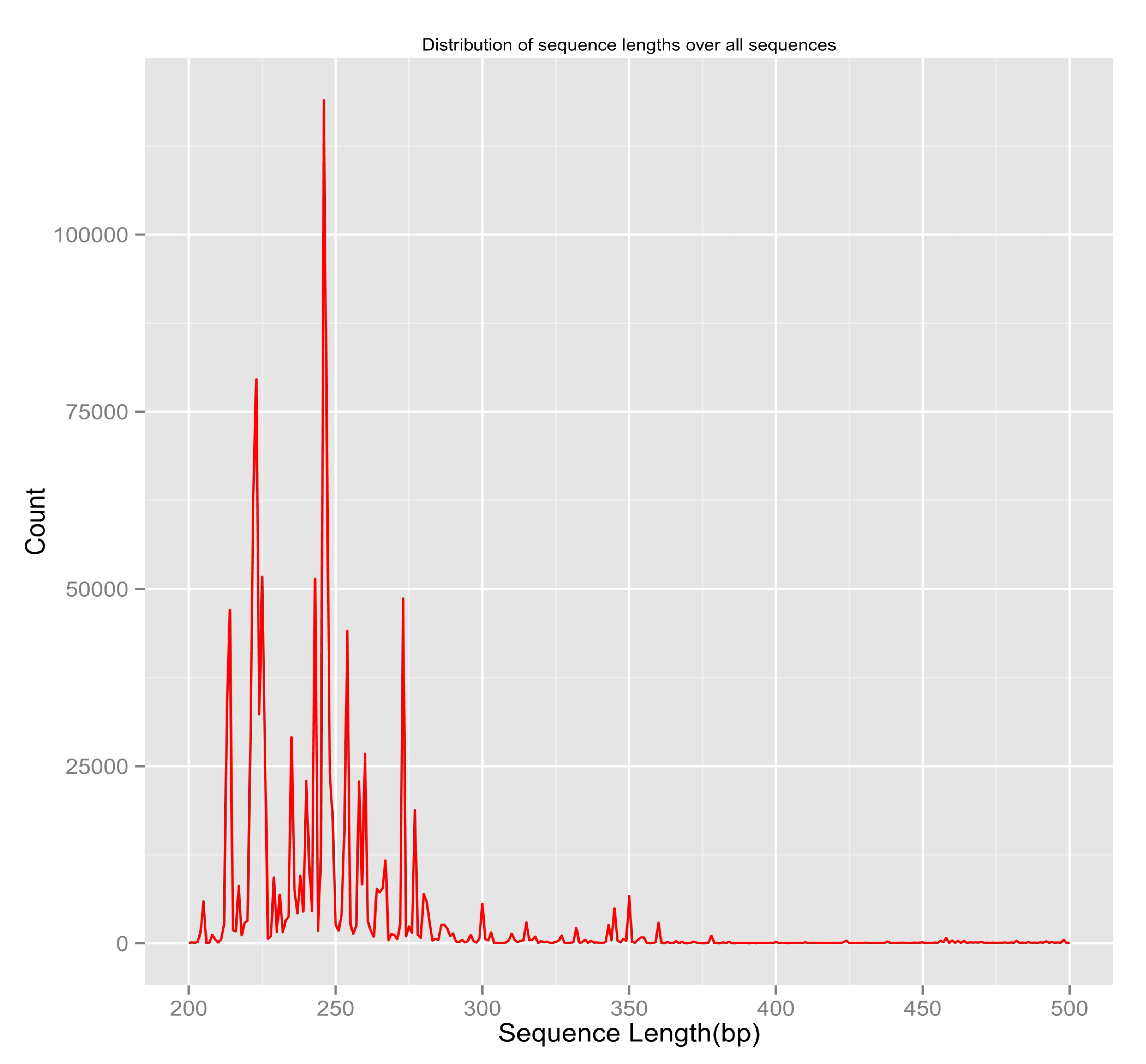


**FIGURE. S2 Soil fungi effective sequence length estimation**


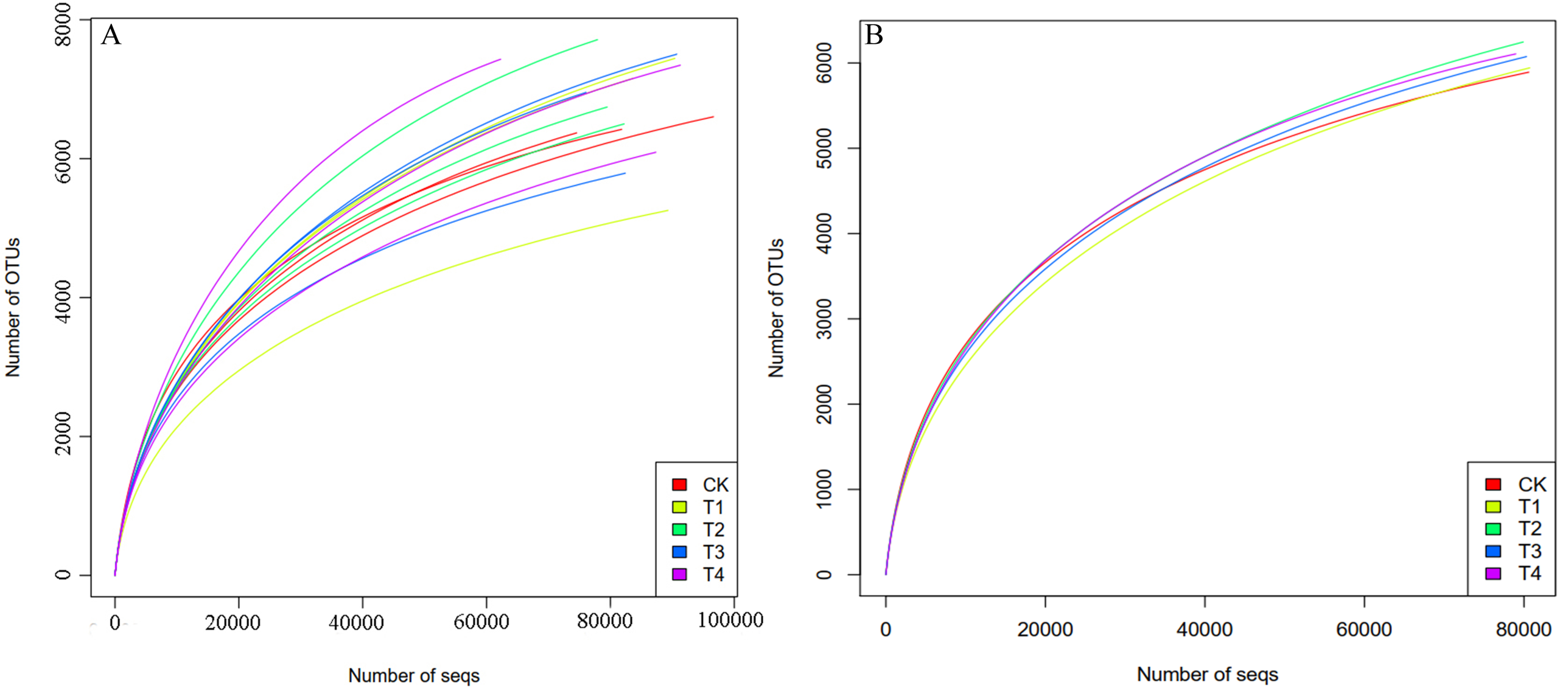


**FIGURE. S3 Dilution curves of soil bacterial samples from different fertilization treatments**

X coordinate for the random number of sequences; Y coordinates of the observed number of OTUs; (a) Dilution curves of soil bacterial samples for each treatment (containing three replicates); (b) overall dilution curves of samples for each treatment. CK: no fertilizer application; T1: conventional fertilizer application; T2: reduced fertilizer application for slow-release fertilizer; T3: conventional fertilizer application for slow-release fertilizer; T4: reduced fertilizer application for conventional fertilizer.


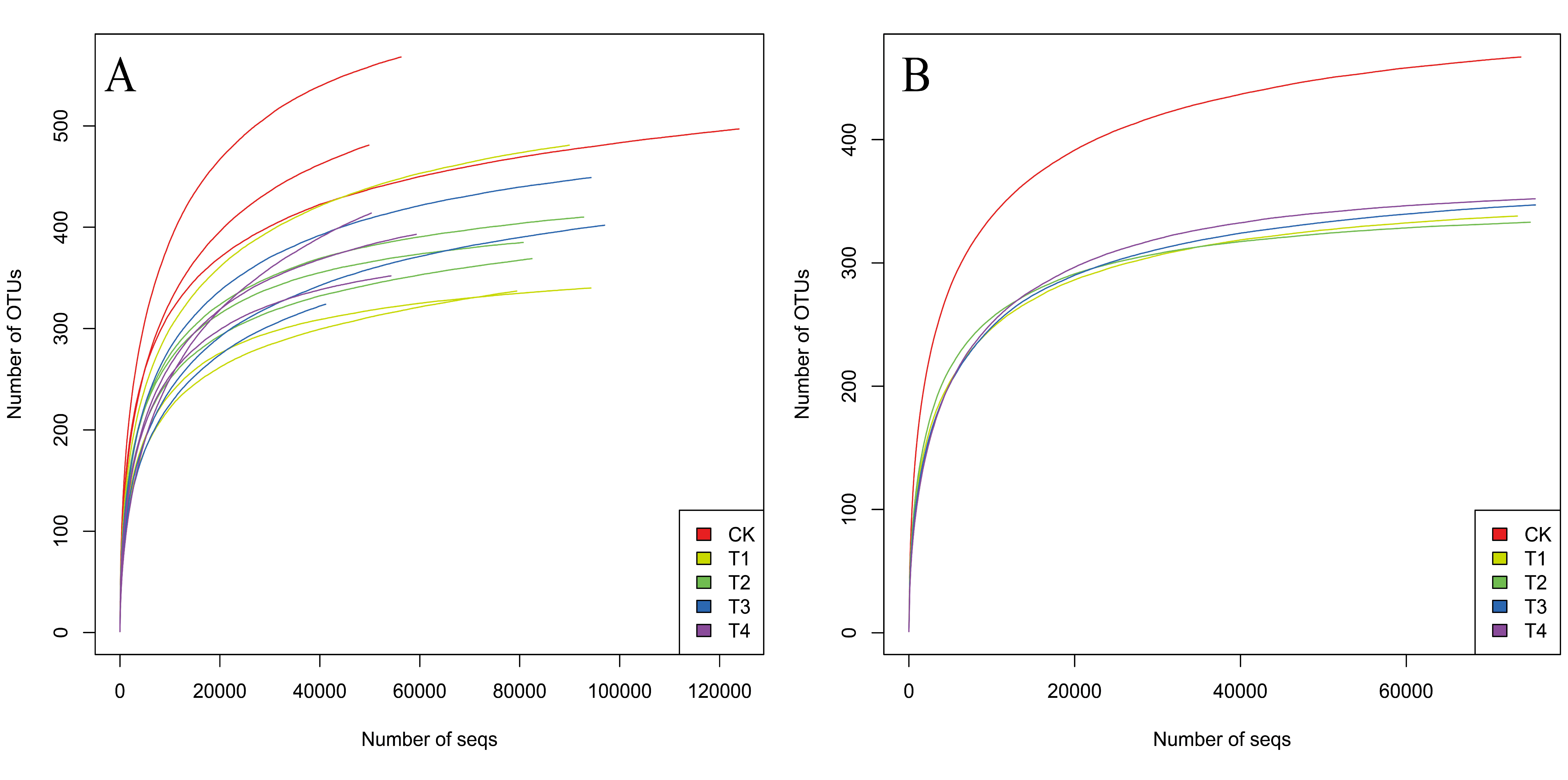


**FIGURE. S4 Dilution curves of soil fungal samples from different fertilization treatments**

X coordinate for the random number of sequences; Y coordinates of the observed number of OTUs; (a) shows the dilution curves of soil fungal samples for each treatment (containing three replicates); (b) shows the overall dilution curves of samples for each treatment. CK: no fertilizer application; T1: conventional fertilizer application; T2: reduced fertilizer application for slow-release fertilizer; T3: conventional fertilizer application for slow-release fertilizer; T4: reduced fertilizer application for conventional fertilizer.
